# Three Pillars of Automated Home-Cage Phenotyping of Mice: Novel Findings, Refinement, and Reproducibility Based on Literature and Experience

**DOI:** 10.3389/fnbeh.2020.575434

**Published:** 2020-10-30

**Authors:** Vootele Voikar, Stefano Gaburro

**Affiliations:** ^1^Neuroscience Center, University of Helsinki, Helsinki, Finland; ^2^Tecniplast S.p.A., Buguggiate, Italy

**Keywords:** mice, phenotyping, telemetry, DVC, IntelliCage, behavior

## Abstract

Animal models of neurodegenerative and neuropsychiatric disorders require extensive behavioral phenotyping. Currently, this presents several caveats and the most important are: (i) rodents are nocturnal animals, but mostly tested during the light period; (ii) the conventional behavioral experiments take into consideration only a snapshot of a rich behavioral repertoire; and (iii) environmental factors, as well as experimenter influence, are often underestimated. Consequently, serious concerns have been expressed regarding the reproducibility of research findings on the one hand, and appropriate welfare of the animals (based on the principle of 3Rs—reduce, refine and replace) on the other hand. To address these problems and improve behavioral phenotyping in general, several solutions have been proposed and developed. Undisturbed, 24/7 home-cage monitoring (HCM) is gaining increased attention and popularity as demonstrating the potential to substitute or complement the conventional phenotyping methods by providing valuable data for identifying the behavioral patterns that may have been missed otherwise. In this review, we will briefly describe the different technologies used for HCM systems. Thereafter, based on our experience, we will focus on two systems, IntelliCage (NewBehavior AG and TSE-systems) and Digital Ventilated Cage (DVC^®^, Tecniplast)—how they have been developed and applied during recent years. Additionally, we will touch upon the importance of the environmental/experimenter artifacts and propose alternative suggestions for performing phenotyping experiments based on the published evidence. We will discuss how the integration of telemetry systems for deriving certain physiological parameters can help to complement the description of the animal model to offer better translation to human studies. Ultimately, we will discuss how such HCM data can be statistically interpreted and analyzed.

## Introduction

Animal models represent a unique source of *in vivo* data for various fields of biomedical research. A recently published summary of statistics from the European Union revealed that between 2015 and 2017 more than nine million animals were used yearly for research purposes in the member states (Report from the Commission to the European Parliament and the Council, 2020). Mice comprise more than 60% of this number. There are several reasons for mice being the most preferred species. The most important factor is the well-known and thoroughly studied genetics of the mice. Different gene targeting techniques are the major tools and methods in modern biomedicine for discovering gene functions and disease mechanisms. Also, the cost-effectiveness and efficiency of mouse studies cannot be underestimated (for example, rapid breeding and smaller animals cost less).

A substantial part of *in vivo* research using mice focuses on the animal locomotor activity as a tool to monitor the animal welfare or to characterize the behavioral profile of the animals for revealing the effects of procedures and manipulations. In such studies, however, some significant variables can remain undervalued: (i) rodents are nocturnal animals, but mostly tested during the light period; (ii) the conventional behavioral experiments take into consideration only a snapshot of a rich behavioral repertoire; and (iii) environmental factors, as well as experimenter influence, are often underestimated.

To run a behavioral core unit, such biases should be considered. Moreover, the need and even demand for novel technology for behavioral analysis have been expressed more than a decade ago (Tecott and Nestler, 2004). An obvious suggestion has been to apply more ethological methods to capture the behavioral repertoire of test animals in their natural environment—the home-cage (Spruijt and Devisser, 2006; Spruijt et al., 2014; Peters et al., 2015).

In the following review, we will discuss the possible solutions for the systematic phenotyping of mouse models by offering a brief overview of technologies available and used for building home-cage monitoring (HCM) systems (summary provided in Table 1). We will then focus our attention on two HCM systems, based on our extensive experience in developing and using these systems. Importantly, the presented solutions can be viewed as additional means for high-throughput phenotyping although not preventing detailed and hypothesis-driven testing. Also, we propose the workflow for longitudinal and continuous monitoring of animals in automated home-cages with the possibility to combine it further with the measure of basic physiological parameters.

**Table 1:** List of commercial systems available for Home Cage Monitoring categorized by platform used.

Technology	System	Animal number	Raw data amount	Outcome parameters	Scalability (number of cages/simultaneous recordings)	Number of publications to date (Google Scholar)
**Video**
	Any-maze Cage (Stoelting)	1 (2 if fur color differs or
dies)	High	Circadian Rhythm Profile, Distance Traveled, Cage
Position	Easy	1,200
	Phenotyper Noldus	1	High	Circadian Rhythm Profile, Distance Traveled, Cage Position, Different Operant walls/Tasks, Fine Behaviors, Food/Water, Running Wheel	Medium	3,560
	Videotrack (Viewpoint)	1	High	Circadian Rhythm, Distance Traveled, Cage Position	Easy	274
	HCA (Actual analytics)	Up to 3 (with RFID)	High	Circadian Rhythm Profile, Distance Traveled, Cage Position, Social Interaction	Medium/Difficult	15
	HomeCageScan (Cleversys)	1	High	Circadian Rhythm Profile, Distance Traveled, Cage Position, Fine Behaviors	Medium	134
**Infrared beams**
	Smart Cage (Omnitech Electronics)	1	Low	Circadian Rhythm Profile, Distance Traveled, Cage Position, Rearing	Medium	1
	Ugo Basile	1	Low	Circadian Rhythm, Distance Traveled, Cage Position, Rearing	Medium	1,650
	AfaSci	1	Low	Circadian Rhythm, Distance Traveled, Cage Position, Rearing, Food and Drinking	Medium	34
	Kinder Scientific	1	Low	Circadian Rhythm, Distance Traveled, Cage Position, Rearing	Medium	135
	Photobeam Activity System (San Diego Instruments)	1	Low	Circadian Rhythm, Distance Traveled, Cage Position, Rearing	Medium	262
	Infrared Motion Detector (Starr Life Technologies)	1	Low	
Circadian Rhythm, Distance Traveled, Cage Position, Rearing	Medium	81
**Sensitive plate**
	Laboras (Metris)	1	Low	Circadian Rhythm, Distance Traveled, Cage Position, Circling Behavior, Fine Behavior	Medium	217
	Activmetre (Bioseb)	1	Low	Circadian Rhythm, Distance Traveled, Cage Position, Wake/Sleep pattern	Medium	3
**RFID**
	Intellicage (TSE)	up to 16 (RFID)	Low	Circadian Rhythm, Different cognition tasks, Water	Medium	117
**Other technologies**
	DVC^®^ Tecniplast	1 or more (depending how many mice are allowed in one cage)	Very low (10 GB/Month for 70 Cages/Rack)	Circadian Rhythms, Distance Traveled (single mouse/cage), Running Wheel	Easy	13

*The second column provided how many animals can be monitored per unit. The third column indicates, on the base of the technology employed, the amount of raw data produced by each system (e.g., large for video and low for infrared beams). The fourth column indicates the parameters that can be extracted based on what is reported in the literature. In the fifth column, it is indicated the scalability of the systems: easy in case video source can be splitted over several subjects/or relative low cost per cage, medium when the purchase of new hardware is needed to monitor additional subjects, medium/high where combination of technologies requires significant investments per unit. In the sixth column it is indicated the number of publications, Google-scholar based, on the search with words combination: “X” indicates the name of the device and company (e.g., DVC Tecniplast), and “home cage,” and mouse, or mice. The patents and references have been excluded from the search. Search concluded on 18th September 2020. Please note that the table is based on our best knowledge provided by the companies’ website or publications content available on google scholar*.

Ultimately, a brief discussion of how to handle HCM generated data statistically would also help to create a sort of guideline for experiments using the systems based on our experience.

## Technologies Used for Monitoring and Recording Animal Behavior Video-Based System

In behavioral neuroscience, video-based analysis systems are still considered the gold standard for many paradigms. The clear advantage is based on the fact that animal behavior is live tracked as well as recorded for later evaluation/assessment (offline). More complex systems (e.g., Phenotyper from Noldus BV) can be used in combination with operant walls to assess performance in complex behavioral tasks. Additionally, recent advancements in artificial intelligence have helped the researchers to recognize natural behavioral states and actions from video-recordings. These can be used as indicators of animal welfare or disturbing behavior. The limitations of such systems are the amount of data produced and storage especially for longitudinal studies as well as the number of animals that can be used in a single unit. Most of the video-based systems apply to single housed animals. However, according to European legislation, this should be avoided. Some systems can distinguish two to four animals with different fur colors (natural or by applying visible or fluorescent dye). One of the limitations or obstacles for proper video recording may be that the cages must normally contain some enrichment items for the proper species-specific environment (e.g., nest material, and shelters), which can hide the animals from the cameras. A combination of video recording with RFID tags has been used by a few systems as an attempt to overcome problems with group housing and cage enrichment. In general, although everyone would like to have evidence for what the animals are doing in their cages (documented by videos), these systems present several limitations regarding the housing of animals, light conditions, camera positions, and importantly, management of large amounts of data.

## Infrared Based System

Recording animal position and activity in space by infrared beams is one of the most traditional methods for automating the behavioral testing. Briefly, an array of infrared beams is surrounding the cage at the animal level, sometimes completed by the second row at a higher level for detecting “vertical” activity (rearing events). Based on the density of the beams, the beam breaks can be interpreted also for finer behavioral outputs (grooming, stereotypic behavior). The major advantage of such technology is the ease of use and the relatively low amount of raw data produced. Therefore, it can be well used for gross circadian rhythm evaluation. However, single housing is always the case here and the duration of monitoring is usually limited to 7 days maximally because of welfare regulations. Moreover, similar to video tracking the infrared systems can be even more vulnerable to problems caused by nesting and bedding material or any other cage enrichment.

## RFID Based System

Individual identification of animals can be feasibly achieved by RFID (radiofrequency identification) transponders (Zeldovich, 2016). These tags are usually implanted subcutaneously (either dorsally or ventrally, depending on the system) under brief anesthesia. The transponders remain passive (no data transmission) until it enters into the electromagnetic field generated by the corresponding RFID antenna. Consequently, it is activated and replies with its unique animal ID number information.

Some RFID based systems leverage this information to uniquely identify the animal when performing a specific task (e.g., occupying the running wheel, or accessing water or eating areas). In this case, this technology works well to facilitate the analysis of single animal behavior in a group-housed situation because reading one animal at a time when approaching the designated area.

Conversely, other RFID-based systems employ an array of RFID antennas entirely mapping the bottom of the area (i.e., the cage) where animals are together. The main goal, in this case, is to track any individual automatically to reconstruct its trajectory while animals are living in a welfare favorable group-housed situation.

This latter design is, unfortunately, prone to more drawbacks because of technological problems. Whenever two (or more) animals are too close to each other, the corresponding RFID antenna located in that area is not capable anymore of reading data because of collision issues between transponders. Moreover, there are more technical issues related to the polling of the array of RFID antennas that cannot be activated all together because otherwise generating cross-talk problems. In the end, the designer of the system has to trade-off between the accuracy of the system and its sensitivity. The more accurate the trajectory would like to be reconstructed, the less sensitivity of reading the system can face, and possibly more missing readings can occur.

Therefore, based on the biological questions being asked, those aspects should be taken into considerations.

## Sensor Plate Systems

A few technologies have been also developed based on sensor plates that detect not only animal basic distance traveled but also more complex behavior such as circling behavior (important for stroke), wake-sleep (active/inactive) patterns. Such systems offer *via* a relatively low data amount a categorization based on the modules that are available in the system and being purchased. The limitation of such systems is that some behaviors remain unclassified and because of missing of the recordings cannot be confirmed. Additionally, only a single animal per system can be used limiting the number of animals that can be studied simultaneously.

## “Do-It-Yourself” Systems

Several groups and laboratories have developed their own equipment based on the combination of above mentioned or additional technologies (Goulding et al., 2008; Shemesh et al., 2013; Genewsky et al., 2017; Balzani et al., 2018; Forkosh et al., 2019; Singh et al., 2019; Anpilov et al., 2020). These systems can be very useful and ingenious for addressing various more or less specific questions related to animal behavior. However, most of this work is carried out by specialized laboratories, and these systems may not be feasible for users in the broader community, especially in core facilities where the balance should be maintained between throughput, training of users, a wide array of questions from different areas of research, etc.

## Can Home-Cage Monitoring Contribute to Enhanced Reproducibility?

The issues of reproducibility in research have been heavily debated during the last decade. For behavioral analysis, the problem is not new—if not before, then since the publication of the seminal article by Crabbe et al. (1999), the issue has been on the table. It is believed and suggested that by using the automated monitoring in the home cage and most importantly, by reducing human bias and interference, the reproducibility can be improved and indeed, quite many supportive evidence exist (Krackow et al., 2010; Robinson et al., 2018; Arroyo-Araujo et al., 2019; Pernold et al., 2019). In the following part, we provide a review of the development and application of two systems where we have substantial hands-on experience within our core/behavioral units.

## Automated Home-Cage Monitoring in Standard Individually Ventilated Cage

**Figure 1:**
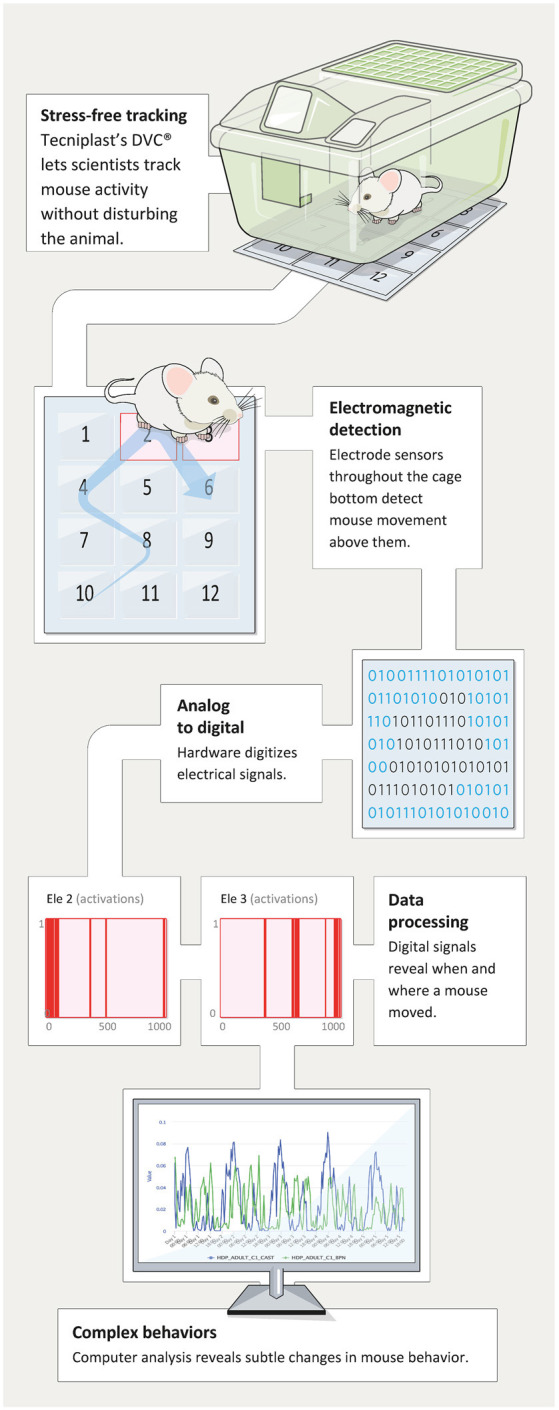
The figure depicts the data flow from racks and mice that *via* moving on the electrode grid on the DVC^®^ boards generate events that then are summarized and displayed through remote access in any browser of choice.

A patented novel solution named DVC^®^ (Digital Ventilated Cage by Tecniplast, shown in Figure 1) has been developed to track the locomotor activity of rodents in near real-time, 24/7 while housed in their home-cage in single- or group-housed conditions. This technology is non-invasive (Iannello, 2019) and proved to be safe both on animal behavior (Burman et al., 2018) and their well-being (Recordati et al., 2019). It can track single animals (distance traveled, velocity) or provide an average percentage of locomotion at the cage level (which corresponds to the average locomotion of single animals). Additionally, the environment in which the animals are tested is the individually ventilated cages (IVC) in which mice are born—a true home-cage environment. This is fundamentally different compared to the other commercially available system where the animals have to be moved in novel, “artificial” cages or testing environments. In fact, it has been shown that changes in the environment (e.g., new olfactory or acoustic cues) or moving to completely new testing device or cage (non-IVC) might produce drastic changes in behavior (Pernold et al., 2019; hyperactivity for 3–4 h) as well as perturbation of the animal’s physiology (e.g., exaggerated heart rate response; Gaburro et al., 2011; Camp et al., 2012).

The DVC^®^ technology is considerably new in the field (available in the facilities for about 5 years) and here we will summarize recent data from this short period. The major limitation of the system is that the group-housed animals cannot be distinguished and therefore the derived data are always considered as an average of the cage and not a singular activity of the animals (discussed further at the end of the review). Therefore, if many animals (e.g., *n* = 4) are in the cage some behaviors can be masked by the average activity of the animals and significant behavioral events might be missed.

## Behavioral Studies

Most behavioral studies on rodents use a battery of behavioral tests that should serve to describe the phenotype(s) of the animals mainly associated with the gene-, environmental- or stress-induced changes mimicking symptoms observed in humans. Such methods are generally based on the assumption that the tested animals have similar baseline locomotor activity. In the open field, a common behavioral test for exploratory activity and locomotion of animals, the main parameter supposed to reflect anxiety-like behavior is time spent and distance traveled in the center of the arena as compared to total activity (Kraeuter et al., 2019). Thus, more anxious animals spend less time in the center of the arena (aversion to the open area) yet total activity should be similar to control animals, otherwise, there is a high probability that the time spent in the center of the arena can be masked or confounded by differences in general locomotor activity.

In the open field, like in other major behavioral tests, most of the variables that could influence the behavior should be controlled, but environmental factors remain an issue in terms of data reproducibility. For instance, a recent multi-center study aimed at large-scale phenotyping using DVC^®^ for HCM demonstrated that even though all factors were controlled (age, sex, breeder, strain, and controlled cage change), the baseline locomotor activity profile of the animals in the three centers differed significantly. The main reason for the unexpected result, despite all the controlled factors, ended up being the environment where the animals were kept and the different schedules of cage change (apparent only after the execution of the experiments; Pernold et al., 2019). In accordance, this article further adds emphasis on what has been reported in the past regarding data reproducibility across labs for *in vivo* preclinical space (review in Kafkafi et al., 2018).

In another study, the researchers aimed at testing the effect of a multi-nutrient diet on recovery in a surgically induced stroke model. Recording the locomotor activity of animals in the HCM-system after inducing the stroke revealed an increase in activity over 3 weeks as an indication of recovery. However, detailed monitoring in the home-cage showed that a special multi-nutrient diet improved the behavioral performance as compared to animals not receiving this diet (Wiesmann et al., 2018; Shenk et al., 2020). Conversely, the same groups of animals exposed to the open field did not display a difference in activity between the 1st and 21st day after surgery. These findings substantiate the fact that testing animals in the home-cage are not only important to gather more reliable scientific results, but also that locomotor activity can serve as a marker to refine surgical practices by improved observation during the recovery period, thus adhering to legislation and the 3Rs principle.

## Metabolic Studies

Research on understanding the brain-gut interaction has gained a lot of interest among the scientific community in the past decade. Especially, transferring the microbiome of patients affected by a specific psychiatric disorder into germ-free animals (animals deprived of their microbiome) and then studying their behavior helps to dissect out molecular mechanisms underlying such pathology (Cryan and O’Mahony, 2011).

In recent work, scientists from Radboud University (NL) used the microbiome of ADHD patients in mice and analyzed the anxiety-related behavior to see whether the high anxiety level present in patients could be reproduced in animals. Baseline locomotor activity measured by HCM-system did not reveal any difference, and that was confirmed by testing animals in an open field, where only a decrease in time spent in the center of the arena (indicative of increased anxiety-related behavior) was revealed. The researchers could then also perform imaging studies to identify brain areas to correlate to the ADHD findings (Tengeler et al., 2020).

## Neurodegenerative Disorders

Neurodegenerative diseases, such as Alzheimer’s or Parkinson’s disease are characterized by behavioral symptoms that are often recognized only in the considerably late phase of the disease progression. Although etiologically different, the two neurodegenerative pathologies share common key symptoms that can be modeled in animals.

One key symptom that can be reproduced is sleep pattern loss. The animal models of Alzheimer’s or Parkinson’s disease show diurnal hyperactivity during the day resembling the patient’s situation of sleep deprivation. The expression of this phenotype can be connected to degeneration in brain areas associated with the regulation of the biological clock.

In fact, in other animal models for neurodegenerative diseases (e.g., amyotrophic lateral sclerosis, ALS), the loss of sleep (or sleep fragmentation) seems to be a preserved symptom. Recently, a mouse model of ALS was characterized using the DVC^®^ technology and the researchers were able to observe that a week before any behavioral manifestations, the sleep pattern measured in the home-cage was already perturbed (Golini et al., 2020). In line with the study, in the same mouse model, using continuous EEG measurement produced a similar observation, corroborating the findings of HCM as well as EEG-based study (Liu et al., 2015; Golini et al., 2020).

## Time Shift Study Change at Circadian Rhythm

For studying the mechanisms of disturbed sleep (e.g., sleeplessness, jet-lag) in animal models, the circadian activity has to be swapped or shifted. Unfortunately, habituation of the animals to a reverse light/dark cycle or a shift is not, so far, objectively measurable. It is commonly believed that mice can swap their circadian rhythms within 2–3 weeks. In recent work, an adaptation of mice to a time shift (from 7 AM-7 PM to 12 AM-12 PM light) in the circadian rhythm was studied. The DVC^®^ system was used to show that all experimental animals changed their locomotor activity within 2 weeks before commencing with the behavioral test in the afternoon time (Goltstein et al., 2018). It is also possible to use the HCM-systems to assess the duration of transition to a new light/dark cycle. The red-colored (non-transparent for mice) cages with an in-cage light- and a time-controlled system called Leddy™ can be used to reverse the light-dark phase of the animals. Animals were placed in the DVC^®^ to check their light/dark inversion objectively. Overall, locomotion patterns could be used to detect how quickly the animals adapt to a change or shift in circadian rhythm (Dauchy et al., 2013).

## Aging Studies

Aging and associated cardiovascular morbidity are research areas that heavily interest the scientific community because of a global aging population. Ongoing studies are showing that as C57BL/6J mice become older, a general overall reduction in the day/night locomotor activity excursions (amplitude) can be observed by HCM. Current preliminary studies are demonstrating that different strains develop subtle different locomotor activity patterns based on their age, genetic background, and environment. In this regard, a large study has been started using outbred mice, recently suggested to be more relevant for the translational purpose (Tuttle et al., 2018), to identify endophenotypes more relevant to the patients (Santin et al., 2020).

## From Baseline Activity Into Complex Behavioral Paradigms

In summary of the first part, the DVC^®^ is not meant for the detection of very subtle behaviors but rather finds its best use in longitudinal studies in which even a small change in disease progression can be identified. For instance, in mouse models of Huntington’s disease and prion disease, the changes in activity as measured in the home cage can be detected already before clinical symptoms (Steele et al., 2007). In the behavioral core facility, the mice are generally tested (depending on the expected phenotype) through a battery of behavioral tests starting from general locomotor activity to more complex behavioral tests addressing several aspects of the putative model. The DVC^®^ (and other HCM-systems) can be seen as suitable tools for establishing and monitoring the baseline activity before and during the behavioral test batteries, but also for detecting the reaction and recovery of animals being exposed to standard tests outside of the home-cage. The acquisition of baseline data can be especially useful when the mice are purchased from any breeder or another institution and subjected to quarantine or general adaptation, usually for 10–14 days before experiments. Therefore, coping-behavior with the new environment (facility), eventual adaptation to the light/dark cycle, human interactions, or other factors that are largely unknown could be addressed already at the cage level. This could help to decide whether the mice need less or more time for adaptation before entering any behavioral experiment.

**Figure 2:**
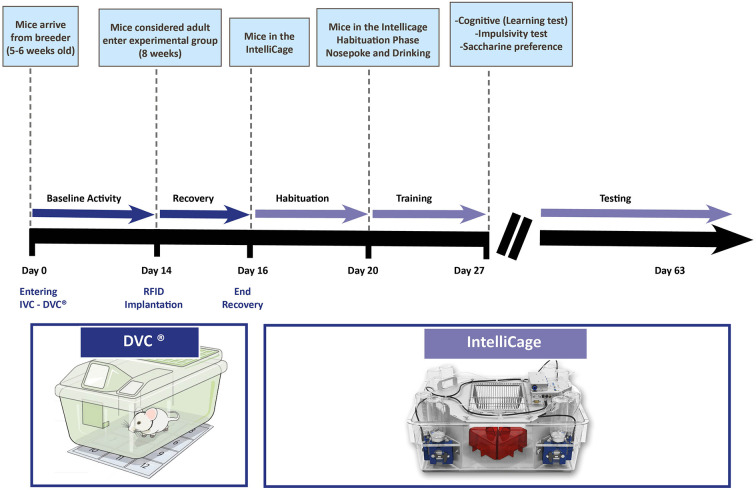
Temporal representation of a hypothetical experimental workflow in a core facility for testing animals with the DVC^®^ system and the IntelliCage. Several phases are incorporated to gather the most of information by combining two systems (bold black line showing the days from the arrival of animals, thinner pink arrow representing the different phases of monitoring—testing in the IntelliCage may contain different protocols for learning, impulsivity, taste preference, stress, et cetera as explained in the text).

Based on our experience, we would like to suggest a workflow (Figure 2) in which the next system, namely the IntelliCage (more detailed presentation in the next section) is introduced. Before starting any experiments on animals, they need time for adaptation in a new facility. This adaptation period can be used for measuring the baseline activity of the animals. If DVC^®^ is complementing the IVC cage, animals can be monitored, and cage activity can be tracked (baseline definition). For individual identification in the IntelliCage, the animals need to be equipped with a radiofrequency identification (RFID) tag (see “IntelliCage” section). After this small procedure and before transfer to the IntelliCage, the animals are monitored for 1–2 days in their home cages to verify that the transponders were not removed or any other side-effects occurred.

## Intellicage

### Basics

**Figure 3:**
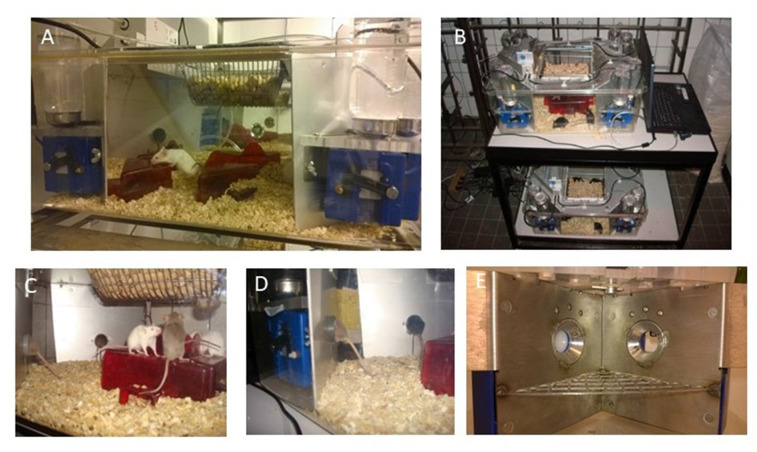
Characteristics and setup of the IntelliCage. **(A)** General view of the IntelliCage and mice in the cage; **(B)** movable version of two cages + laptop on a trolley; **(C)** mice standing on the shelters and reaching the food; **(D)** mice entering the corners; and **(E)** inner view of the conditioning corner (note two holes for nose poke, closed on the left and open on the right side, with nipple visible there).

IntelliCage (shown in Figure 3) is a brand name for the system which allows automated monitoring of behavior in group-housed mice (or rats) for long periods (IntelliCage by NewBehavior, TSE-Systems, Germany). Importantly, no human interference (handling of animals) is needed during experiments. This is a rather unique system among other home-cage solutions. In this system, the mice can be maintained in groups while many different behavioral or cognitive characteristics can be tested without removing animals from the cage. This contrasts with many other systems where single housing is required and/or only recording of spontaneous behavior is feasible. The properties comparing the advantages and limitations of different systems are presented and discussed elsewhere (Richardson, 2015; Bains et al., 2018).

The roots of the IntelliCage are in the field station for ecological and comparative brain research, which was located in the western part of Russia (Dell’Omo et al., 2000; Lipp et al., 2001). Transponder technology was applied for detecting the activity and learning ability of small rodents in naturalistic settings (Dell’Omo et al., 2000; Vyssotski et al., 2002). Based on these experiments, the downscaled version of the outdoor pen was created (Galsworthy et al., 2005). Just 1 year before this publication, Tecott and Nestler (2004) expressed concern that “Optimal use of the rapidly escalating numbers of mouse lines engineered for these purposes (elucidating pathophysiology and treatment of neuropsychiatric diseases) is hindered, however, by practical and theoretical limitations of common behavioral analyses” and called for new strategies combining automated behavioral monitoring and information technologies.

The hardware of the system consists of four conditioning corners. In each of these corners, the animal can make a nose poke to the hole on the left or right side and access the nipple of the bottle for drinking. Importantly, these holes can be blocked by doors, and opening requires certain conditions to be met. Thus, everything is based on instrumental conditioning.

The setup is not a home-cage in its literal meaning. The animals need to be transferred to the new cage, which is substantially larger than the “normal” home-cage in the colony room. However, in most cases, the same type of bedding and enrichment material as in colony housing can be used. One advantageous difference from the other HCM systems is that the cages can be flexibly moved in the facility (see Figure 3). It is possible to keep up to 16 mice in the large cage (the floor area available for mice is 1,612 cm^2^, the required minimum for mice over 30 g is 100 cm^2^). In the case of female mice, the groups can be formed just before testing, whereas for male mice it is recommended to keep the group smaller (eight animals) and to form the group as early as possible after weaning to avoid aggression between the subjects (based on personal communications and experience). Another procedure before starting the experiment is the injection of transponders (RFID-tags) subcutaneously (on the back). The procedure is done under brief anesthesia.

RFID-tags are needed for individual recognition—only one animal at a time can enter the conditioning corner. Based on the animal number, the events and actions in the corner are controlled. The sensors of the hardware record the following events: (1) visits (entering the corner—start, end, duration); (2) nose pokes (number, side, duration); and (3) licks (number and duration of tongue contacts with nipple). The IntelliCage has four corners, each with two sides, and each corner and side can be defined as correct, neutral, or incorrect. Eventually, there are several possibilities to create a design for the behavioral tasks—from simple place (corner) preference to more complex patrolling designs, but also to measure the aspects of attention, impulsivity, taste preference, etc. One of the main limitations of using the IntelliCage is that animals are detected only if they enter the corner, thus the general activity outside the corners is missing.

### Adaptation to the Cage

As the mice are transferred to a novel space (larger cage, conditioning corners) containing familiar items (bedding, enrichment), some time is needed for adaptation. Briefly, during the adaptation phase, the mice can freely enter all corners where both doors are open—there is no restriction for drinking (this is called the free adaptation period). The most important procedural goal to achieve before continuing with more demanding cognitive tasks is that animals will learn to enter the corners and drink there. However, data collection can be started immediately, and the first hours and days provide valuable information about neophobia, exploratory activity, spatial preference and stereotypy, circadian, and other spontaneous patterns of activity. It has been shown that behavioral profiles created using individual component scores were highly characteristic for different inbred strains or different lesion models of the nervous system. Therefore, careful analysis of the adaptation period of 7 days can contribute significantly to the high throughput prescreening of mutant mice (Vannoni et al., 2014). The next phase (nose poke adaptation) is a step towards operant conditioning—the doors in the corners are closed, the door will be open only upon the first nose poke at the respective hole. The door remains open for 5–10 s (programmable)—this is a time allowed for drinking during a given visit, to open the door and drink again the animal has to leave the corner and start a new visit.

### Learning, Memory

Learning always requires some motivation. Protocols for conventional tests of learning and memory (e.g., radial arm maze, T-maze) often involve food deprivation, followed by using the food as a reward during training and testing. In IntelliCage, water (drinking) is a reward, therefore the learning tasks are carried out during drinking sessions. The drinking session means that animals have most typically two slots during their active (dark) period when the doors in the corners can be opened by nose pokes. The mice adapt very quickly to such timing (temporal conditioning) and can be maintained on this regime for a long time without any detectable problems for welfare (Voikar et al., 2018). It has been shown that mice tolerate water deprivation much better than food deprivation (Tucci et al., 2006). Another obvious improvement and refinement are that learning sessions can always be conducted during the active period of the animals, regardless of the actual light cycle in the facility.

There are several possibilities for applying the place learning tasks. The simplest version is to make water accessible only in one corner of the cage, followed by reversal (rewarding the opposite corner). However, this appears to be an easy task even for mice with a hippocampal lesion (Voikar et al., 2018), although some models show impaired or slower acquisition. It is important to remind that not all mice in the cage do not need to be trained to visit the same corner, it is better to counterbalance the rewarded corner. Learning to visit the same corner may lead to group learning which can be another interesting feature for testing. For instance, it has been shown that transgenic mice modeling Alzheimer’s disease was capable to learn normally when co-housed with wild-type control mice. However, impaired learning became evident when the transgenic and control mice were housed in separate cages (Kiryk et al., 2011). The system allows easy implementation of complex and challenging learning tasks requiring sequential visits to different corners during drinking sessions to get access to water. These tasks are called patrolling, chaining, or flexible sequencing (Endo et al., 2011; Kobayashi et al., 2013; Voikar et al., 2018). We have shown that a bilateral lesion to the hippocampus does not affect simple place preference, whereas tasks that are more complex revealed gradually impaired performance, depending on the task difficulty (Voikar et al., 2010, 2018).

In addition to positively rewarded (appetitive) learning it is also possible to apply punishment in the IntelliCage for incorrect actions (visit, nose poke, and lick). For this purpose, an air-puff is used as a negative reward (Voikar et al., 2010). Importantly, this is not a noxious stimulus and therefore has much better compliance with the 3Rs principle (compared to several other behavioral tests where for instance electric foot-shocks are applied).

### Impulsivity

Assessment of impulsivity has been challenging in mouse models. The most popular method is the five-choice serial reaction time task (Robbins, 2002). However, this task is characterized by a lengthy training period (several weeks to months) before testing is possible, moreover, food restriction, and single housing are often applied. We have developed the protocols for motor impulsivity and delay discounting in the IntelliCage (Kobayashi et al., 2013; Mätlik et al., 2018). These designs provide a substantial refinement to conventional methods.

### Testing Taste and Addiction-Related Behavior

As licking (drinking) is one of the actions recorded, and one cage can contain up to eight drinking bottles (two in each corner) it becomes obvious that the system has a great potential in measuring the gustatory functions (taste preference) which otherwise need to be carried out by a two-bottle choice test (Patrikainen et al., 2014). Also, the intake of alcohol and other substances can be controlled and combined with behavioral tasks (Radwanska and Kaczmarek, 2012; Smutek et al., 2014; Koskela et al., 2018).

### Social Behavior

Maintenance of animals in social groups for behavioral tasks can be viewed as either a potential for discoveries or a limitation for interpretation of the results. Undoubtedly, the interaction between mice of different genotypes will affect their behavior, as has been shown for inbred strains and disease models (Kiryk et al., 2011; Heinla et al., 2018). With special design and custom analysis, it is feasible to get insight into the social behavior of the mice (Kulesskaya et al., 2013; Nowak et al., 2013; Puścian et al., 2014; Smutek et al., 2014).

### Studies on Stress

Finally, an excellent set of experiments (resembling chronic unpredictable mild stress) has been conducted for studying the mechanisms of depression, the action of antidepressants, and the role of the environment (Branchi et al., 2013a,b; Alboni et al., 2016, 2017). It is good to keep in mind here and in general that the IntelliCage represents an enriched environment (social interaction, large space, conditioning corners, and procedures). The effect of environmental enrichment to alleviate, reverse, or delay pathological symptoms in mouse models is well known (van Dellen et al., 2000). Therefore, the interpretation of phenotypic differences between conventional tests and IntelliCage should be done cautiously.

### Standardization and Reproducibility

The last decade in science has been heavily influenced by a “reproducibility crisis.” For behavioral neuroscience, one of the landmark articles to open the discussion about reproducibility and standardization was published in 1999 (Crabbe et al., 1999), where the authors showed systematic differences in mouse behavior across three laboratories despite extensive standardization. Since then, the need and meaning of standardization for animal research have been debated (Würbel, 2000, 2002; van der Staay and Steckler, 2002; van der Staay et al., 2010; Crabbe, 2016; Voelkl and Würbel, 2016) along with some solutions offered (Richter et al., 2010; Kafkafi et al., 2018; Voelkl et al., 2020). Not very surprisingly, it was convincingly shown that one of the major factors contributing to the variability of data could be the experimenter (Chesler et al., 2002; Bohlen et al., 2014) and handling methods (Gouveia and Hurst, 2017), along with the autonomic stress-response displayed by mice when handled and placed in novel arenas (van Bogaert et al., 2006). Therefore, one could hypothesize that reproducibility can be enhanced by reducing the handling and human interference during the experiments. Several studies carried out during validation of the IntelliCage confirmed this idea—consistent strain differences were detected in multiple laboratories when similar procedures were applied (Krackow et al., 2010; Endo et al., 2011; Codita et al., 2012). Extreme standardization of the environment is not possible; moreover, it is against the principle of external validity of basic research. However, the standard versions of commercially available HCM-systems are the same in each laboratory, collecting the data in a standardized manner. Therefore, these data are comparable between the laboratories and not affected by human observer nor by differences in equipment.

### Combination of Behavior and Physiological Parameters

The integration of behavior and physiology has become more feasible year by year. A quick search in PubMed with keywords “Animals AND Physiology AND Behavior” revealed a constant increase from 1984 (first telemetry probe produced) up to 2018 in the number of publications (up to 600.000).

In animal behavior, most of the studies focus on identifying and understanding the neuronal correlates of a specific behavioral repertoire. However, in the stage of a behavioral test battery where the goal is to phenotype the rodents for their genetic changes or drug effects, this is mostly not applicable. In high throughput behavioral research, the most applied technology is telemetry because animals can be studied in their home-cage as well as long-term studies, without interfering with animal behavior. Several studies proved that the implantation of a telemeter did not affect behavioral performances as compared to control animals. More importantly, those telemeters (e.g manufactured by DSI—Data Sciences International, TSE-Systems, Emka Technologies) provide recordings of the most commonly used physiological stress markers: heart rate, blood pressure, and body temperature. Those markers have been extensively studied in models of post-traumatic stress disorder (PTSD; Gaburro et al., 2011; Camp et al., 2012), depression, and neurodegenerative diseases (Kuzdas et al., 2013). In particular, heart rate variability, which assesses the variation of the heart rate/time and can be calculated in several ways, has proved to be far more sensitive than behavioral outputs to provide insights about disease progression before behavioral manifestations or responses to a specific drug for proper dosage (Stiedl and Meyer, 2003; Gaburro et al., 2011; Vandendriessche et al., 2014; Agorastos et al., 2019). These approaches can be highly translatable to humans. However, the major limitation of such an assessment is the complexity and quantity of data. Therefore, good tools for analysis are required for handling such an amount of data for reasonable interpretation. In a typical behavioral battery, telemetric characterization can sometimes represent a challenge because either the telemeter does not cover a long-range and therefore impact the arena performances, or on the other side do not warrant a continuous (24/7) signal needed to fully characterize the behavior. Additionally, synchronization of several systems is mostly needed (through TTL signals or other output) to warrant that behavioral changes can be correctly assigned to the specific change in the physiological marker currently studied.

Overall, the telemetric assessment in a behavioral unit would be mostly assigned to study whether the genetic modification or the drug to be studied is potentially having an unspecific behavioral outcome on the one side (e.g., changes in sleep patterns, seizures) or where long-term effects have to be evaluated (neurodegenerative diseases). However, telemetry is not performed as a standard procedure due to the complexity of data, invasiveness, and cost.

### Statistical Analysis of HCM Data—Facility-Based Experience

The problem of single or group housing of animals, especially for behavioral neuroscience experiments, has become a serious trade-off between welfare and the scientific result (Nagy et al., 2002; Martin and Brown, 2010; Kappel et al., 2017; Jirkof et al., 2020). The findings suggest that depending on biological factors (sex, age, strain) the different housing conditions (single vs. group) can have a substantial effect on the phenotype and therefore it should always to be considered in study design and interpretation (Voikar et al., 2005; Kulesskaya et al., 2011; Lander et al., 2017). Especially in male mice, the social hierarchy, aggressive behavior, and fighting may play an important role and hurdle (Lidster et al., 2019). However, it has been shown that male mice prefer the social proximity independent of their social rank (van Loo et al., 2004), therefore the decision should be made case-by-case and weighed against the scientific objectives to be tested (Kappel et al., 2017).

Therefore, even if limited by confounding factors (uncontrolled social interaction of group-housed animals) but promoted by welfare obligations, we would like to briefly address how the data from in-between subject indistinguishable behaviors (“cage behavior”) or single (RFID tagged animals) distinguishable animals’ behavior(s) in group-housed conditions could be statistically analyzed. The problem of identifying the experimental, observational, and biological units for analysis should be carefully considered in each individual study by researchers and reviewers (Lazic et al., 2018).

One of the main advantages of home monitoring systems is that the mice can be observed for a long period, allowing researchers to perform aging studies or to assess the evolution of slow diseases (Golini et al., 2020). This kind of studies needs proper statistical testing for repeated measures, that can be parametric (Repeated Measures ANOVA, provided the parametric assumptions are not violated) or non-parametric like a nonparametric test for longitudinal data (nPARLD) ANOVA type statics (ATS; Noguchi et al., 2012). Linear mixed-effects models are also a good tool for modeling and testing longitudinal data, especially with many repeated measurements. This is important if there is the issue of missing data (which can easily occur during long experiments). In the framework of a repeated measure, *post hoc* analyses require a proper and careful approach. The conventional Bonferroni correction is often too conservative for strongly correlated repeated measures and a large number of comparisons, possibly increasing the number of false-negative results. Alternatives have been proposed in the literature, for example, Dubey and Armitage-Parmar (D/AP) Procedure (Sankoh et al., 1997) and different versions of False Discovery Rate (Benjamini and Yekutieli, 2001).

Another issue that is worth considering is the reduction in sample size because of group housing. As stated before, the DVC^®^ system can measure the aggregated level of activity, when mice are placed in a group, and not the individual behavior of each mouse. Consequently, each cage is considered as one subject, or experimental unit, independent of the number of animals in the cage) which differs from the number of biological or experimental units (Lazic et al., 2018). The sample size (N) is therefore reduced, even though it does not necessarily scale down exactly with the aggregation factor, due to the intra-cage correlation (Barcikowski, 1981). Animals housed together in the same cage may show similar behavior and this should be considered also in “traditional” studies where mice, after being housed together in multiple cages, are considered as the experimental units for the analysis, potentially leading to incorrect results (Basson et al., 2020).

Additional future studies should investigate how to evaluate the adequate sample size in DVC^®^-like studies, where cages are the units of analysis, based on the level of the dependency intra-cage, the required statistical power, and the effect size.

Regarding the Intellicage or RFID-based similar systems, provided the data to be analyzed following the statistical parametric test assumptions, as the mice are uniquely identified with the RFID antenna placed e.g., at the corners, according to study design, and analysis of variance with covariates test could be utilized to assess the asked hypothesis. Statistically speaking, the sample size is given by the animal (experimental units equal to biological and observational units). However, to limit the dependency of the animal to another, due to the social environment and for study design, one should consider additional mixed groups (e.g., mixing treated and control animals as a third cage) for better testing of the question being asked. Importantly, should also animal activity be uniquely tracked with RFID antenna and without video, because of previously mentioned RFID technology limitation, the readings/data are more prone to violate the assumptions of parametric data (i.e., data dependency). In this case, the cage (not the animal) is considered as an experimental unit. Therefore, a similar analysis of those explained for the DVC^®^ could be employed. In general, conventional behavioral testing often does not consider the time as a factor (e.g., animals tested at a different time of the day/season; Chesler et al., 2002) or that the animals are in groups before entering a behavioral test and shortly after (according to baseline), therefore home-cage base activity around the standard behavioral test could aid to take account for unwanted events across groups.

### Automated Monitoring to Help Facility Management at Critical Times—Example of Covid-19

Although by law [Directive 2010/63/EU on the protection of animals used for scientific purposes (Parliament, 2010)], the health status of animals has to be checked daily, there may be situations when this is complicated. Recently, several articles reported the reduction and culling of the animal colonies because of the lockdown applied due to the Covid-19 pandemic (Nowogrodzki, 2020; Pullium, 2020). This is still a problem as experiments have to restart from the beginning and throwback experimental research for several years. In the first place, each animal facility needs to have a crisis management plan, which would help to meet and deal with such situations. Good management combined with HCM could significantly alleviate the burden. In this respect, Tecniplast carried out a survey (in 12 facilities) regarding contingency management during Covid-19 time, to understand if and what alternatives are considered to culling. It appeared that almost 50% of the participants managed to organize their work in shifts instead of culling the colonies. Also, more than half of the participants would consider a video or alternative system to help with the workload while limiting the exposure of the personnel to unwanted risks (Gaburro, 2020).

If the vital parameters (drinking, feeding, anomalies in locomotor activity as an indicator of animal welfare) can be detected remotely, it would be possible to reduce the personnel entering the animal facilities during critical times. Moreover, monitoring of environmental parameters and cage characteristics can add flexibility for the timing of cage changes and performing other care-taking activities. Thus, at least some time could be gained for planning further steps instead of immediately culling the colonies. Additionally, the power of running the study and continuous collection of the data without the presence of an experimenter cannot be underestimated.

## Summary

Animal research is still an important part of basic biomedicine and studies on physiology and behavior in live animals are increasing (note—the number of animals has remained rather stable over the last years, but new technology allows more efficient and versatile use of animals). Efforts are being made to ensure and enhance animal welfare, the principle of 3Rs, scientific and translational validity. Technology for monitoring the animals and measuring behavior is developing very rapidly and we believe that these advancements will contribute to achieving the goals mentioned above. However, having an all-in-one system is probably too complicated and idealistic—each development will have pros and cons, strengths, and limitations. In this review, we provided evidence for the application of two different HCM systems for mouse phenotyping, based on published literature and personal experience. We suggest that comprehensive long-term monitoring will substantially contribute to enhancing the scientific validity of the experiments, as this could eventually offer the best way to evaluate for expression and/or progression of symptoms and endophenotypes of disease models.

## Author Contributions

VV and SG wrote the manuscript together. All authors contributed to the article and approved the submitted version.

## Conflict of Interest

SG works at Tecniplast S.p.A. as Scientific Director. The remaining author declares that the research was conducted in the absence of any commercial or financial relationships that could be construed as a potential conflict of interest.
